# Evaluating and Regulating Lead in Synthetic Turf

**DOI:** 10.1289/ehp.1002239

**Published:** 2010-10-01

**Authors:** Gregory Van Ulirsch, Kevin Gleason, Shawn Gerstenberger, Daphne B. Moffett, Glenn Pulliam, Tariq Ahmed, Jerald Fagliano

**Affiliations:** 1 Agency for Toxic Substances and Disease Registry, Atlanta, Georgia, USA; 2 New York State Department of Health, Troy, New York, USA; 3 Department of Environmental and Occupational Health, University of Nevada Las Vegas, Las Vegas, Nevada, USA; 4 New Jersey Department of Health and Senior Services, Trenton, New Jersey, USA

**Keywords:** Child care settings, expert panel, interim standardized approach, potential lead exposures, uncertainties

## Abstract

**Background:**

In 2007, a synthetic turf recreational field in Newark, New Jersey, was closed because lead was found in synthetic turf fibers and in surface dust at concentrations exceeding hazard criteria. Consequently, public health professionals across the country began testing synthetic turf to determine whether it represented a lead hazard. Currently, no standardized methods exist to test for lead in synthetic turf or to assess lead hazards.

**Objectives:**

Our objectives were to increase awareness of potential lead exposure from synthetic turf by presenting data showing elevated lead in fibers and turf-derived dust; identify risk assessment uncertainties; recommend that federal and/or state agencies determine appropriate methodologies for assessing lead in synthetic turf; and recommend an interim standardized approach for sampling, interpreting results, and taking health-protective actions.

**Discussion:**

Data collected from recreational fields and child care centers indicate lead in synthetic turf fibers and dust at concentrations exceeding the Consumer Product Safety Improvement Act of 2008 statutory lead limit of 300 mg/kg for consumer products intended for use by children, and the U.S. Environmental Protection Agency’s lead-dust hazard standard of 40 μg/ft^2^ for floors.

**Conclusions:**

Synthetic turf can deteriorate to form dust containing lead at levels that may pose a risk to children. Given elevated lead levels in turf and dust on recreational fields and in child care settings, it is imperative that a consistent, nationwide approach for sampling, assessment, and action be developed. In the absence of a standardized approach, we offer an interim approach to assess potential lead hazards when evaluating synthetic turf.

## Background and Statement of Issues

In 2007, the New Jersey Department of Health and Senior Services (NJDHSS) and the Agency for Toxic Substances and Disease Registry (ATSDR) investigated a scrap metal facility in Newark, New Jersey. During the investigation, experts from NJDHSS and ATSDR observed children playing on an adjacent athletic field. Because lead was one of the contaminants at the facility, NJDHSS collected samples from the field to test for lead that might have migrated from the facility to the field. Lead was identified in dust (3,742 mg/kg) and turf fibers (3,500 mg/kg) collected from the field. Because lead concentrations exceeded federal and New Jersey hazard criteria for lead in dust and soil, the field was closed as a prudent public health response (ATSDR 2008). Public health professionals across the country began testing synthetic turf and evaluating whether it represented a lead hazard.

On 18 June 2008, the Centers for Disease Control and Prevention (CDC) issued a Health Alert, recommending testing of fields that are made from nylon or nylon-blend fibers and have fibers that are abraded, faded, or broken, or contain visible dust. Through observation and data review, it is evident that synthetic turf fibers can degrade from use (wear) and exposure to ultraviolet radiation and other atmospheric conditions. This degradation can produce fragments of broken fibers and turf-derived dust on the field [see Supplemental Material, Figure 1 (doi:10.1289/ehp.1002239)]. Persons who play or work on aging turf fields may be exposed to lead in the dust. For children, exposure would most likely occur by ingestion through hand-to-mouth contact. The Health Alert also issued general guidelines to reduce the potential for children’s exposure to dust generated from synthetic turf degradation. On 30 July 2008, the U.S. Consumer Protection and Safety Commission (CPSC) released an analysis of synthetic turf and concluded that young children are not at risk from exposure to lead on these fields (CPSC 2008).

Because there are many existing environmental lead standards, differences between regulatory agency opinions, and various approaches to testing lead in synthetic turf, we lack standardized approaches for sampling synthetic turf and interpreting the results to assess potential lead hazards. Moreover, according to some experts, a thorough understanding of the exposure and health risks associated with lead in turf is needed (Lioy and Weisel 2008). Given this backdrop, our purpose in this commentary is to

- Increase awareness of potential lead exposure from synthetic turf by presenting current data, especially new data that show elevated lead in fibers and dust from turf used in child care settings
- Identify uncertainties that complicate the risk assessment process
- Recommend that federal and/or state agencies, with input from other interested parties, systematically address and determine appropriate methodologies for assessing lead in artificial turf
- Recommend an interim standardized approach for sampling, interpreting results, and taking health-protective actions.

Chromium as well as lead may be present in synthetic turf because lead chromate (PbCrO4) pigment is used in turf fibers. Based on atomic weight differences, the concentration of chromium in turf fibers should be about four times lower than the concentration of lead. Although in this commentary we focus on lead, there are potential health concerns associated with exposure to chromium. Measures to limit possible lead exposures at turf fields also will help to reduce exposures to chromium.

## Results of Turf Sampling and the CPSC Study

To evaluate the type(s) of synthetic turf that are most likely to contain elevated concentrations of lead, we conducted or reviewed several investigations. These studies include data collected from the following investigations and from data provided by the CPSC investigation.

Table 1 shows results of testing of synthetic turf fibers in four areas, including limited dust sampling results: *a*) Data from NJDHSS include 13 recreational fields and 4 new products (residential/landscaping application) [ATSDR 2008; Hudson Regional Health Commission (HRHC) 2008; NJDHSS 2008]; *b*) data from New York State are from a high school—one recreational field (Life Science Laboratories 2008); *c*) data from New York City, New York are from four recreational fields [New York City Department of Health and Mental Hygiene (NYC DHMH), unpublished data]; and *d*) data from a U.S. Army Base, South Korea are from one recreational field (U.S. Army, unpublished data).

Bioaccessibility testing performed by NJDHSS in 2008 confirmed that lead in evaluated synthetic turf products became soluble under simulated digestive conditions (NJDHSS 2008). Gastric bioaccessibility from turf fibers ranged from 14.5% to 50.9% (Table 1, surfaces 1 through 3). Gastric bioaccessibility was highest in the synthetic-based dust sample at 92.2% (Table 1, surface 3) (NJDHSS 2008).

Tables 1 and 2 show results of synthetic turf fiber and dust-wipe sampling at the University of Nevada, Las Vegas (UNLV), on 10 surfaces in children’s play areas in child care settings or on athletic fields (UNLV, unpublished data). UNLV also performed soil analysis in areas adjacent to the fields to identify possible non-turf sources of lead. UNLV used U.S. Environmental Protection Agency (EPA) risk assessment protocols to conduct soil and dust-wipe testing. To provide a realistic approximation of lead concentrations in the dust, we assigned a value of limit of detection (LOD)/2 to all samples below the LOD. We also present data with samples below the LOD removed (Table 2). Assigning all samples below the LOD a value equal to the LOD would likely overestimate potential exposures.

Compiled data (Table 1) show the testing results for synthetic surfaces and new products. Twelve of 29 actively used synthetic surfaces and two of four new turf products tested exceeded the statutory lead limit of 300 mg/kg for consumer products intended for use by children [Consumer Product Safety Improvement Act (CPSIA 2008)] and the U.S. EPA lead hazard standard of 400 mg/kg for residential soil (U.S. EPA 2001). Turf fibers exceeding the statutory limit included polyethylene, nylon, and polyethylene/nylon blends. Results indicate elevated lead concentrations predominantly in green nylon-based turf fibers and polyethylene-based turf fibers of various colors.

UNLV wipe-sampling results ranged from below the LOD to 81 μg/ft^2^, which exceeded U.S. EPA’s hazard standard for floors, 40 μg/ft^2^ (U.S. EPA 2001) [Tables 1 and 2; also see Supplemental Material, Figure 2 (doi:10.1289/ehp.1002239)]. Wipe sampling results were available for six surfaces (surfaces 22–26, and 28; Table 1) which exceeded the CPSIA lead limit of 300 mg/kg (CPSIA 2008), with one surface exceeding the U.S. EPA lead dust hazard standard for floors, 40 μg/ft^2^. Background testing indicated soil-lead concentrations that were all < 20 mg/kg. Additionally, three New Jersey fields showed lead loading greater than the U.S. EPA lead-dust hazard standard (U.S. EPA 2001).

The CPSC tested lead in turf fibers from four manufacturers/vendors (CPSC 2008). Concentrations ranged from nondetectable to 9,600 mg/kg. Using a wipe-sampling method developed for testing pressure-treated wood, CPSC sampled eight fields (five identified as “in use”) from the four manufacturers. The reported years of installation of the fields ranged from 1999 (one field) to 2008 (two fields).

The CPSC’s guidance policy advises that exposures of > 15 μg lead/day may result in blood-lead levels of 10 μg/dL in children (CPSC 1998); the CPSC used this guidance policy for sample comparison. The average of three dust-wipe samples from the oldest field, containing an average of 5,500 mg/kg lead in turf fibers, was 68 μg/400 cm^2^ wipe area (i.e., 158 μg/ft^2^). The CPSC divided the wipe-sample results by five to approximate the amount of wiped material that may transfer to children’s hands. The CPSC also assumed that children transfer 50% of the lead on their hands into their mouths. All the lead intake estimates were < 15 μg/day, and CPSC concluded that young children are not at risk from lead exposure at synthetic fields (CPSC 2008). However, for the oldest field tested, CPSC estimated an average lead intake of 6.8 μg/day. This estimation supports our supposition that as turf fields age, they can deteriorate and increase the risk of cumulative lead exposures to children.

## Turf Sampling Methods and Public Health Comparison Values

Determining the concentration of lead in or on synthetic turf can include measuring the concentration of lead in fibers (results expressed as mass of lead per mass of fiber tested), the concentration of lead in the dust formed from fiber degradation (results expressed as mass of lead per mass of dust tested), and/or surface dust-lead loading (results expressed as mass of lead per area of surface sample). The kind of sampling required depends on the information being sought. Each type of sampling has advantages and disadvantages (Table 3).

Qualified persons (e.g., U.S. EPA–certified lead risk assessors) should conduct sampling. State or local health and/or environmental agencies also may be able to provide guidance for sampling, such as field locations that should be tested and types of equipment to use. In states with environmental laboratory approval programs, approved or certified laboratories should perform sample analyses. Otherwise, a laboratory accredited by the National Environmental Laboratory Accreditation Conference should conduct the analyses.

Health-based values or standards developed specifically for evaluating lead in synthetic turf have not been established. However, values developed for other media can be used to assess the potential health significance of synthetic turf-sampling results. These values are included in the CPSIA of 2008, which established the statutory limit for lead in consumer products intended for use by children (300 mg/kg) and the U.S. EPA hazard standards established for lead in soil (400 mg/kg and 1,200 mg/kg in bare residential soil in children’s play areas and yard-wide average, respectively) and dust on floors (40 μg/ft^2^, including carpeted floors) (U.S. EPA 2001). These values and their potential applicability to synthetic turf-sampling results are shown in Table 4. Other relevant comparison values include the CDC’s guidelines for blood-lead levels in children (action level > 10 μg/dl) (CDC 1991) and the CPSC’s guidance policy for lead exposure in children (exposure > 15 μg/day) (CPSC 1998).

## Synthetic Turf Assessments, Health Implications, and Actions

With the possible exception of sweep sampling, all of the sampling methods shown in Table 3 have been used by different entities to sample synthetic turf surfaces. These entities evaluated potential lead hazards by assessing lead loading on turf surfaces, lead concentration in turf fibers, lead concentration in dust generated by degrading turf, or a combination of these measures. Assessments also included estimates of lead intakes and blood lead levels. Actions that were taken based on these assessments included no action, access restrictions, temporary closure, and field or surface replacement.

CDC publications about reducing childhood lead exposures recommend that government agencies develop and apply systematic approaches to prevent exposures to even small amounts of lead in consumer products, identify and correct lead hazards, and develop and implement strategies that encourage the elimination of lead hazards, including the use of safer alternatives (CDC 2007). The CDC recommends the elimination of all nonessential uses of lead. To address these CDC recommendations, the synthetic turf industry has voluntarily agreed to reduce lead levels in accordance with the CPSIA of 2008 (Synthetic Turf Council 2008). Further, the August 2009 Consent Judgment, resulting from the California Proposition 65 action against several synthetic turf businesses, prohibits those businesses from selling certain synthetic turf products in California if the products contain > 100 mg/kg of lead (Consent Judgment as to Astroturf, Inc. 2009). This Consent Judgment could mean that in other states as well, synthetic turf containing reduced lead levels will be sold.

The potential concern addressed in this commentary is for turf that has already been installed, especially in children’s play environments. Our findings and those presented in the CPSC study indicate that synthetic turf can deteriorate over time to form dust containing lead at levels that may pose a risk to children who play on these surfaces [Supplemental Material, Figures 1 and 2 (doi:10.1289/ehp.1002239)] (CPSC 2008; HRHC 2008; NJDHSS 2008; UNLV, unpublished data). Based on the findings presented, further study should be conducted to determine the extent of lead exposure, specifically to children, that synthetic turf products pose. Also, given new data that showed elevated lead levels in turf and dust in child care settings (UNLV, unpublished data), a consistent, nationwide approach for sampling, assessment, and action is needed.

## Specific Recommendations and Conclusions

Significant issues include the absence of a standardized approach for sampling synthetic turf, using sampling results to assess potential exposures and lead hazards, and identifying appropriate health-protective actions. In the absence of a standardized approach, the interim approach outlined as follows could be used. This approach is derived from the sampling methods and public health comparison values shown in Tables 3 and 4. An expert panel, convened by federal and/or state agencies, could help provide guidance on a standardized, nationwide approach.

- Determine the lead concentration of synthetic turf fibers. If the concentration is > 300 mg/kg, perform surface-wipe testing to determine the surface dust-lead loading. If the concentration is < 300 mg/kg, further action is unnecessary. This value is proposed because it is the statutory limit established by the CPSIA of 2008 for the lead content of consumer products intended for use by children (effective 14 August 2009). In addition, surface-wipe sampling is proposed because this kind of sampling may best represent accessible surface lead to which people may most readily be exposed (Bai et al. 2003).
- If the wipe-testing results show lead loadings > 40 μg/ft^2^ and young children or other susceptible individuals (e.g., pregnant women) are likely to have frequent/prolonged contact with the turf (e.g., child care settings), restrict access of children and susceptible individuals and replace the turf with turf containing lead concentrations < 300 mg/kg as soon as practical. This loading value, the U.S. EPA hazard standard for floors (U.S. EPA 2001), is proposed because it was developed to protect young children for a surface where a relatively high potential for lead exposure exists.
- If the wipe-testing results show lead loadings > 40 μg/ft^2^ and young children or other lead susceptible individuals (e.g., pregnant women) are not likely to have frequent, prolonged contact with the turf (e.g., athletic fields where older children or adults are primary users), restrict access of young children and susceptible individuals and replace the turf with turf containing lead concentrations < 300 mg/kg as soon as practical.

Implementing the dust-sampling component of this approach should be given higher priority for synthetic turf that has fiber-lead concentrations > 300 mg/kg and is in poor condition, which is indicated by widespread areas of wear and/or visible fiber dust. Information reviewed in this commentary demonstrates that turf-generated dust may approach lead dust hazard criteria in 2–4 years in some cases. Therefore, synthetic turf that has fiber-lead concentrations > 300 mg/kg, but is in good condition, should be monitored routinely for signs of wear and dust generation, with dust sampling being initiated as determined by professionals trained in lead hazard identification.

To date, no study has linked turf exposures to elevated childhood blood lead levels. However, physicians should be aware of synthetic turf as one potential source of exposure for young children, especially given its use in residential, child care, or other play environments. Health officials investigating elevated blood lead in children should also be aware of synthetic turf as a potential source of lead exposure.

## Figures and Tables

**Table 1:** Total lead concentrations in turf fibers and in surface dust from tested synthetic surfaces: sampling period August 2007 through September 2008.

Tested surface	Use	Fiber type^d^	Year installed	Turf fiber^a^ lead concentration (mg/kg)	Lead dust loading^b^ (μg/ft^2^)^c^	
					Detected values	All data
New Jersey^e^						
1	Recreational	Nylon	1999	3,500	49 (*n* = 6)	49 (*n* = 6)
2	Recreational	Nylon	2003	3,400	65^f^ (*n* = 4)	45^f^ (*n* = 6)
3	Recreational	Nylon	1999	4,100	4,047 (*n* = 1)	4,047 (*n* = 1)
4	Recreational	Polyethylene	2003	< 1.4	NA	NA
5	Recreational	Polyethylene	2003	< 2.1	NA	NA
6	Recreational	Polyethylene	2007	< 1.0	NA	NA
7	Recreational	Polyethylene	2006	< 1.7	NA	NA
8	Recreational	Polyethylene	2005	< 1.4	NA	NA
9	Recreational	Polyethylene	2007	1.4	NA	NA
10	Recreational	Polyethylene	2005	2.0	NA	NA
11	Recreational	Polyethylene	2006	< 1.4	NA	NA
12	Recreational	Polyethylene	2007	< 1.0	NA	NA
13	Recreational	Polyethylene	2003	1.6	NA	NA

New York^g^						
14	Recreational	Nylon (yellow)	1999	4,500	NA	NA
14	Recreational	Nylon	1999	4,900	NA	NA
14	Recreational	Nylon (white)	1999	36	NA	NA
14	Recreational	Nylon (red)	1999	140	NA	NA
14	Recreational	Nylon (blue)	1999	42	NA	NA
15	Recreational	Nylon	1998	3,202–5,284	14.1 (*n* = 5)	14.1 (*n* = 5)
16	Recreational	Nylon	1998	< 16.2–159.7	ND	< 8.3 (*n* = 3)
17	Recreational	Polyethylene	2001	84.9–353.2	ND	< 8.3 (*n* = 4)
18	Recreational	Polyethylene	2006	< 15.3–< 16.4	12.6 (*n* = 1)	5.84 (*n* = 5)

South Korea^h^						
19	Recreational	Polyethylene	2007	1,400	NA	NA
19	Recreational	Polyethylene (yellow)	2007	1,500	NA	NA
19	Recreational	Polyethylene (red)	2007	630	NA	NA
19	Recreational	Polyethylene (white)	2007	21	NA	NA

Nevada^g^						
20	Recreational	Polyethylene nylon	2000	85	23.33 (*n* = 3)	11.18 (*n* = 4)
21	Child care facility	100% Nylon	2007	ND	NA	NA
22	Child care facility	100% Nylon	2005	5,100	ND	10 (*n* = 6)
23	Child care facility	100% Nylon	2004	8,800	ND	10 (*n* = 2)
24	Child care facility	100% Nylon	2004	5,700	42.75 (*n* = 12)	37.53 (*n* = 15)
25	Child care facility	100% Nylon	2006	6,400	35 (*n* = 2)	26.67 (*n* = 3)
26	Landscape	100% Nylon	2005	6,200	29.77 (*n* = 44)	29.77 (*n* = 44)
27	Child care facility	Polyethylene nylon	2004	94	ND	10 (*n* = 6)
28	Child care facility	Polyethylene nylon	2004	1,100	ND	10 (*n* = 9)
29	Recreational	Polyethylene nylon	2004	25	ND	10 (*n* = 13)

New product						
31	Residential	Polyethylene nylon	NA	1,000	NA	NA
31	Residential	Polyethylene fraction	NA	< 0.97	NA	NA
31	Residential	Nylon fraction	NA	3,500	NA	NA
32	Residential	Nylon	NA	4,700	NA	NA
33	Recreational	Polyethylene	NA	< 0.92	NA	NA
34	Recreational	Polyolefin (infill material)	NA	< 0.92	NA	NA

Abbreviations: NA, no data available; ND, not detected.

a Turf fiber testing: analytical methods: EPA 3050B/6010B, for surfaces 1–13, 19, 31–34; EPA 3050B/7420 for surfaces 14–18, 20–29. Testing highlights synthetic fibers that exceed the CPSIA (2008) lead standard for consumer products of 300 mg/kg (effective 14 August 2009).

b Lead dust loading testing: sampling and analytical methods: ASTM method D7144-05a EPA 3050B/6010B for surface 1; HUD guidelines EPA 3050B/7420 for surfaces 2, 15–18; EPA SOP 2040 (modified) EPA 3050B/6010B for surface 3 [see Supplemental Material, Figure 1 (doi:10.1289/ehp.1002239)]; NIOSH (National Institute for Occupational Safety and Health) 9100/7082 for surfaces 20–29. Testing highlights lead dust loading values that exceed the U.S. EPA lead dust hazard standard for floors (U.S. EPA 2001).

c Mean lead concentration.

d Green turf fibers unless otherwise noted.

e Composite sample (green turf fibers) collected from nine locations from the playing surface of turf fields: surfaces 2–13.

f HRHC 2008.

g Samples below the limit of detection (LOD) were assigned a value of half the LOD to demonstrate the number of samples collected compared with those that contained detectable concentrations of lead: surfaces 15–18, 20–29.

h U.S. Army Testing of Polyethylene Turf Demonstrates Lead at 1500 mg/kg (U.S. Army, unpublished data).

**Table 2:** Descriptive data for soil, turf, and dust collected at child care and recreational surfaces in Clark County, Nevada, 2008.

	Mean	Median	Mode	SD	Minimum	Maximum	*n*
Dust^a^,^b^	32.18	24	20	17.5	20	81	61
Dust^a^,^c^	20.4	10	10	16.18	10	81	132
Soil^b^	10.41	10	10	3.28	7	18	17
Soil^c^	9.66	10	10	3.83	3	18	19
Turf^b^	3,353	3,100	10	3,402	10	8,800	10
Turf^c^	3,048	1,100	3.5	3,382	3.5	8,800	11

Abbreviations: Max, maximum; Min, minimum. Data are from Table 1, surfaces 20–29. Samples below the LOD were assigned a value of half the LOD to demonstrate the number of samples collected compared with those that contained detectable concentrations of lead.

a LOD ranged from 10–20 μg/cm^2^ depending on sample size.

b Summary of samples that tested above the LOD.

c Summary of all samples collected.

**Table 3:** Sampling methods for evaluating lead in or on synthetic turf.

Purpose of sampling	Medium sampled	Sampling method	Advantages	Disadvantages	Considerations
Determine lead concentration in turf fibers (mg/kg or equivalent)	Turf fibers	Bulk sample of turf fibers	Simple Can determine if turf has potential to pose a lead hazard in the absence of reliable information from the turf manufacturer/vendor	Does not yield useful exposure information because people are unlikely to ingest intact turf fibers	Not all synthetic turf fibers contain lead Different colored fibers should be tested separately. Predominant color should be given priority

Determine lead concentration in dust (mg/kg or equivalent)	Fiber dust	Sweep	Simple Data are relevant for assessing exposure	May be difficult to collect adequate mass for laboratory analysis	

		Micro vacuum (e.g., ASTM Method D7144-05a) (ASTM 2005)	Data are relevant for assessing exposure	Some potential for overestimating exposure. Vacuum sampling is reliable for assessing total lead contamination in carpets but may capture embedded dust that is not readily accessible for exposure (Bai et al. 2003).	Other considerable methods (Bai et al. 2003; Farfel et al. 1994; Farfel et al. 2001; Lanphear et al 1995; Lioy et al. 2002; Sterling et al. 1999).
		High-volume vacuum (e.g., U.S. EPA’s Standard Operating Procedure 2040) (U.S. EPA 2002)			

Determine lead loading on surface area (μg/ft^2^ or equivalent)	Fiber dust	Surface wipe	May be superior for ease of use, cost and standardized quality control procedures for laboratory analysis (Sterling et al. 1999). Has been recommended for measuring “accessible lead” (lead to which people are readily exposed) (Bai et al. 2003). Consistent with the U.S. EPA requirements for evaluating dust lead hazards on surfaces, including carpeted surfaces, in residential dwellings (U.S. EPA 2001).	May yield variable results (Sterling et al 1999).	Some household dust studies indicate that dust lead loading is more predictive of children’s blood lead levels than is dust lead concentration (Sterling et al. 1999). Dust lead loading can be measured by vacuum or wipe sampling. Both have been widely used for residential sampling and at synthetic turf fields (ATSDR 2008; HRHC 2008; NYC DHMH, unpublished data). The same kinds of vacuums used for concentration measurements also can be used for loading measurements
		Micro vacuum	Has been recommended for obtaining information on total lead accumulation (Bai et al 2003).	Some potential for overestimating exposure (Bai et al. 2003).	A commonly used wipe sampling method is described in U.S. Department of Housing and Urban Development guidelines on lead-based paint hazards in housing (HUD 1995).
		High-volume vacuum			

ASTM, American Society for Testing and Materials.

**Table 4:** Public health comparison values potentially applicable for evaluating synthetic turf sampling results.

Synthetic turf medium	Comparison value	Purpose for which value was developed	Potential applicability to synthetic turf	Limitations
Turf fibers	300 mg/kg	Statutory limit on lead content of consumer products intended for use by children^a^	To determine if turf fibers contain lead at levels that may warrant dust sampling	Frequency/duration of exposure to turf is likely less than for toys and other children’s consumer products
Turf dust	400 mg/kg	Hazard standard for lead in bare residential soil (children’s play areas) (U.S. EPA 2001)	To determine if the concentration of lead in dust poses a potential lead exposure hazard	Standards designed to be protective of young children in a residential setting
	1,200 mg/kg	Hazard standard for lead in bare residential soil (yard-wide average) (U.S. EPA 2001)		Greater likely potential for exposure to soil/dust in a residential setting than to dust on synthetic turf
	40 μg/ft^2^	Hazard standard for lead in dust on floors (including carpeted floors) (U.S. EPA 2001)		40 μg/ft^2^ standard may be more reasonable for turf applications such as in indoor play areas or child care centers where children may have prolonged exposure, than for athletic fields where children are less likely to have frequent, prolonged turf contact Turf measurements greater than the hazard standard do not necessarily suggest a hazard comparable to an exceedance of the standard in a residential setting

a CPSIA (2008). As of 10 February 2009, the standard was 600 mg/kg. Effective14 August 2009, the standard was reduced to 300 mg/kg. Effective 14 August 2011, the standard will be further reduced to 100 mg/kg unless the CPSC determines that 100 mg/kg is not technologically feasible for a product or product category
